# Socioeconomic differences in adolescent health behaviors and their effect on inequalities in adult depressed mood: findings from a 27-year longitudinal study

**DOI:** 10.1186/s12888-025-06679-6

**Published:** 2025-04-10

**Authors:** Magnus Jørgensen, Bente Wold, Otto R.F. Smith, Ellen Haug

**Affiliations:** 1 Department of Health Promotion and Development, University of Bergen, Bergen, Norway; 2 Department of Health Promotion, Norwegian Institute of Public Health, Bergen, Norway; 3 Department of Teacher Education, NLA University College, Bergen, Norway; 4 Department of Public Health, University of Stavanger, Stavanger, Norway; 5 The Environmental Health Institute, University of Lisbon, Lisbon, Portugal

**Keywords:** Life course epidemiology, Depressive symptoms, Adolescence, Health behaviors, Lifestyle psychiatry, Socioeconomic status, Socially differential vulnerability, Socially differential exposure

## Abstract

**Background:**

Health behaviors have been posited to partly explain the association between socioeconomic status (SES) and health (i.e., the behavioral explanation of health inequalities), yet few studies have examined whether health behaviors serve as pathways from adolescent SES to adult depressive symptoms. This study aimed to explore the effects of adolescent health behaviors on adult depressed mood using the adolescent pathway model (APM).

**Methods:**

Our sample consisted of *n* = 1109 Norwegians [45.5% female], who were surveyed from ages 13 to 40 across ten time points. Using linear regression analyses, we examined (1) the association between parental SES [household income and parental education] and adolescent health behaviors [breakfast regularity, leisure time physical activity (LTPA), difficulties falling asleep, alcohol consumption, and smoking], and (2) the associations between adolescent health behaviors and adult depressed mood, and whether these were moderated by indicators of parental SES. We also assessed how health behaviors are associated with social inequality in adult depressed mood. In this context, social inequality was defined as the covariance between adult SES (i.e., income and education) and adult depressed mood.

**Results:**

Higher household income predicted higher levels of LTPA, and higher parental education predicted greater breakfast regularity. None of the health behaviors were associated with adult depressed mood, nor did they show moderation by SES. Adolescent health behaviors did not independently account for social inequality in adult depressed mood.

**Conclusions:**

The study suggests minimal socioeconomic differences in adolescent health behaviors, which do not significantly account for social inequalities in adult depressed mood. This offers limited support for the behavioral explanation of health inequalities within the framework of the APM. However, adolescent depressed mood emerges as the strongest predictor of adult depressed mood, highlighting its importance as a key focus for early intervention efforts.

**Supplementary Information:**

The online version contains supplementary material available at 10.1186/s12888-025-06679-6.

## Introduction

Social inequalities in depression are apparent across age groups [1–3], and the differences begin to emerge already in adolescence and young adulthood when depressive symptoms begin to have their onset [4]. According to behavioral theories, these inequalities stem from differences in health behaviors [5]. That is, adolescents (and adults) from lower socioeconomic groups are posited to engage more in risky and harmful health behaviors that, in turn, are associated with poorer health outcomes. Notably, health behaviors (i.e., physical activity, sleep, diet, etc.) have mostly been studied in relation to physical health outcomes, however, in recent decades, lifestyle psychiatry has emerged as an important field linking lifestyle factors to mental health [5]. The increasing interest in this field is, in part, due to the fact that health behaviors are highly adaptable and offer promising opportunities for low-cost interventions at an early stage [6]. This is also evident in the increased policy efforts towards promoting adolescent health behaviors (e.g., physical activity, breakfast consumption, sleeping etc.) to reduce the risk of long-term health issues [7, 8].

The lifestyle psychiatry research field, which explores the links between lifestyle factors and psychiatric outcomes, such as depression [9], is supported by several empirical studies which link a diverse range of health behaviors to depressive symptoms. For instance, studies indicate that skipping breakfast increases odds of depression and adverse mental health outcomes [10]. Similarly, it has been found that higher physical activity levels in adolescents are linked to lower levels of adult depressive symptoms [11, 12]. Among various types of sleeping issues in adolescence, difficulties falling asleep (i.e., sleep onset latency) - the most common sleep problem for adolescents [13] - has also been clearly linked to depression in longitudinal studies [14]. More harmful health behaviors, such as alcohol consumption [15] and smoking [16] have similarly shown both direct and bidirectional effects with depressive symptoms, but across shorter periods of time. However, to what extent these health behaviors are associated with socioeconomic differences in depressive symptoms is less clear.

**Fig. 1:**
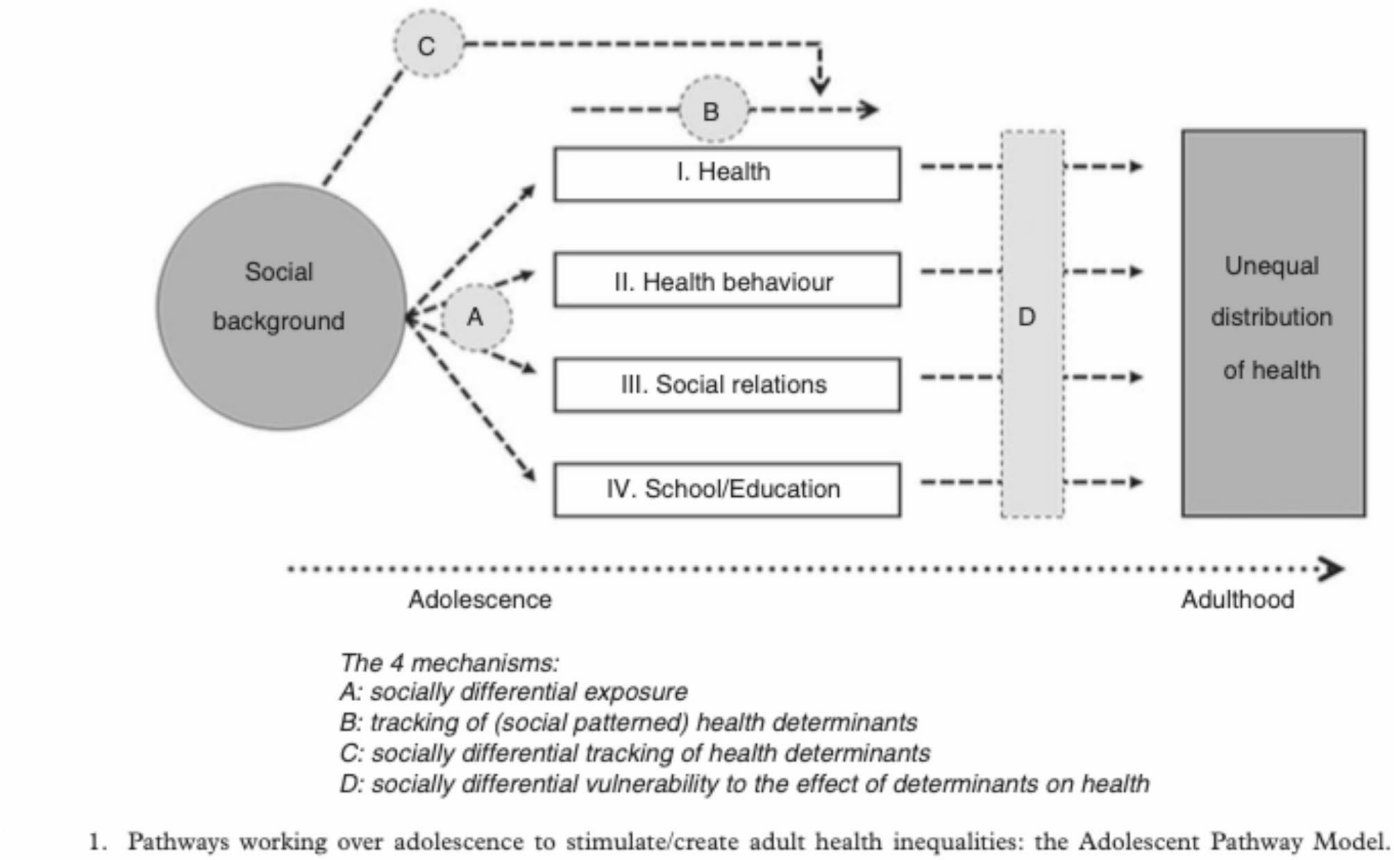
The adolescent pathway model. **Note.** Figure showing the Adolescent Pathway Model. From “Pathways and mechanisms in adolescence contribute to adult health inequalities” by Due, P., Krølner, R., Rasmussen, M., Andersen, A., Trab Damsgaard, M., Graham, H., & Holstein, B. E., 2011, *Scandinavian Journal of Public Health*, 39(6_suppl), 62–78. Copyright 2011 by the Nordic Societies of Public Health

The Adolescent Pathway Model (APM) (See Fig. 1) provides a valuable perspective on this issue, proposing that adolescent health behaviors are one of several pathways linking parental socioeconomic background to health disparities in adulthood (i.e., the behavioral explanation for health inequalities) [17]. Within the APM, two key mechanisms are particularly important: first, certain pathways to health inequalities (i.e., health behaviors) may be distributed unevenly across socioeconomic groups (socially differential exposure); second, the impact of these pathways may vary among (socioeconomic) groups (socially differential vulnerability). These mechanisms align with the behavioral explanation of health inequalities, as they emphasize how socioeconomic context modifies the effects of health behaviors on health disparities in adulthood [5]. More specifically, the APM asserts that socioeconomic differences in health behaviors emerge as adolescents from lower socioeconomic backgrounds engage in more detrimental health behaviors than their higher SES peers (Mechanism A in Fig. 1) [18, 19]. This mechanism has been empirically supported by several studies on health behaviors [18–27]. This also includes studies on skipping breakfast [28], smoking [29], sedentary behavior [30], and poor sleeping patterns [31]. As adolescents grow older, these differences are assumed to persist (Mechanism B in the APM) and show adverse development for lower SES peers compared to peers from higher socioeconomic backgrounds (Mechanism C in the APM) [17].

As a result, by the time individuals reach adulthood, the prolonged accumulation of stressors - known as allostatic load [32] - combined with limited resources such as lower social support and poorer finances [33, 34], may lead to dysfunctional stress regulation [35] and heightened stress sensitivity [36]. In support of this, empirical studies show that detrimental and harmful health behaviors are linked to increased levels of allostatic load as well as higher levels of depressive symptoms [37]. This, in turn, can contribute to impaired immune function [38], diminished psychological well-being [39], and overall deteriorated health [40]. Collectively, these factors may create a socially differential vulnerability to depressed mood and other health issues in adulthood, as illustrated in Mechanism D of Fig. 1. Importantly, low levels of beneficial health behaviors, such as regular physical activity, adequate sleep, and a healthy diet, are also relevant in this context, as they may further exacerbate vulnerability by failing to mitigate the effects of stress and supporting overall resilience.

Among the mechanisms in the APM, socially differential exposure has received the most empirical attention while socially differential vulnerability has received the least [17]. Thus, it remains unclear whether adolescents from lower SES backgrounds exhibit greater long-term vulnerability to developing depressive symptoms in adulthood due to their health behaviors during adolescence. One of the few empirical studies that have looked at this is a longitudinal study from the UK, which examined clusters of risky and less-risky health behaviors in mid-adolescence and their association with psychological distress at age 42, using parental occupational ranking as a moderator [41]. The study found no evidence of moderation by parental SES, which could be due to the choice of SES indicator, because occupational classes tend to be heterogeneous, with significant variations in income and educational background even within the same occupation [42]. In contrast, parental education is considered a more precise indicator of lifestyle and behavior-related factors [43]. Similarly, household income offers a direct measure of a family’s economic access to material resources (e.g., food, housing), which can significantly influence health behaviors and their impact on depressive symptoms [44, 45]. Relatedly, these considerations highlight the importance of distinguishing between SES indicators, as education, income, and occupation have been shown to have low to moderate correlations [46]. Using separate indicators allows for a clearer understanding of each indicator’s impact, while a composite index might obscure these distinct influences [42].

Beyond socially differential vulnerability, the role of health behaviors in explaining the social gradient in adult depressive symptoms remains unknown. That is, how much of the association between adult depressive symptoms and adult socioeconomic indicators (such as educational level and income) can be attributed to adolescent health behaviors? Earlier research has predominantly focused on the relationship between health behaviors and inequalities in mortality and cardiometabolic outcomes [25, 47]., and few, if any, studies have considered adolescent health behaviors’ contribution to social inequalities in adult mental health as implied by behavioral explanation of SES-based health inequalities.

To address the gaps and limitations in the literature, this study aimed to examine how adolescent health behaviors influence adult depressed mood within the APM framework. Specifically, the study explored three key research questions: [1] To what extent do key adolescent health behaviors display socioeconomic differences (socially differential exposure) [2]? Are adolescents from lower socioeconomic backgrounds more vulnerable to developing depressed mood in adulthood based on health behaviors during adolescence (socially differential vulnerability) [3]? To what extent do adolescent health behaviors explain the relationship between adult depressed mood and adult education and income?

Depressed mood was chosen as the mental health outcome due to its significant impact on major life domains (e.g., work, family) even before reaching clinical thresholds [48]. As a cardinal symptom of depression [49], depressed mood is a less ambiguous [50] and highly relevant marker for studying mental health outcomes. The key adolescent health behaviors examined—breakfast regularity, leisure-time physical activity (LTPA), sleep difficulties, alcohol consumption, and smoking—were selected based on their established links to both socioeconomic background and depressive symptoms, as shown in previous studies [10, 13–16, 28–31, 51–53]. Socioeconomic background was measured using two primary SES indicators: parental education and household income.

## Methods

### Sample

The present study used data from the Norwegian Longitudinal Health Behavior Study (NLHBS) (*n* = 1242), which began in 1990. An initial total of 1195 adolescents aged 13 years were sampled from randomly selected schools in Hordaland County, Norway, and agreed to participate. Respondents were subsequently followed up at nine time points (1991, 1992, 1993, 1995, 1996, 1998, 2000, 2007, and 2017). Participants’ parents were also surveyed on health-related variables and socioeconomic indicators in 1990 (*n* = 948), 1993 (*n* = 600), and 1996 (*n* = 622). The Data Inspectorate of Norway reviewed the study, and the Regional Committee of Medical Research Ethics (REK) approved it. All participants provided informed written consent for the collection of their data for the NLHBS at each time point. Response rates are described elsewhere [54]. Of the total sample (*n* = 1242), 63.37% had dropped out by the final time point in 2017. An earlier attrition analysis revealed that individuals still participating in 2017 were more likely to be female and have parents with higher levels of education and household income [55], suggesting some selection effects in the sample. An additional attrition analysis on health behaviors is also provided in the results section of the present study.

### Instruments

#### Outcome measure

The Depressive Tendencies Scale by Alsaker [56] was used to measure depressed mood by having respondents rate seven items from 1 = does not apply at all to 6 = applies exactly. A mean score for depressed mood was calculated, based on measurements at ages 13, 15, and 18 (1990, 1992, 1995) for adolescent depressed mood, and at age 40 (2017) for the adult outcome measurement. For each occasion, a minimum of four items was required to compute the mean score. An earlier survey wave using the present scale and the Center for Epidemiological Studies Scale for Depression showed good concurrent validity (*r* =.82) [57]. Partial longitudinal scalar invariance and satisfactory reliability have also been established in an earlier study on the present scale [55].

### Health behaviors

Breakfast regularity was measured at ages 13, 14, 15, 18, and 19 with the item: “During a normal week, how often do you eat breakfast?” Response options ranged from 1 = not that often to 4 = every day. Leisure-time physical activity (LTPA) was measured at ages 13, 14, 15, 16, 18, and 19 with the item: “Outside of school/work: How often do you engage in sports or physical exercise to a degree that is exhaustive or makes you sweat?” Responses were reported on a 7-point scale, ranging from 1 = none to 7 = every day. Difficulties falling asleep were measured with the item: “Have you had difficulties falling asleep during the last three months?” at ages 13, 14, 15, 16, and 19. Answer categories ranged from “seldom or never” to “more than four evenings per week.” Alcohol consumption was measured at ages 14, 15, 16, 18, and 19 with the item: “How often have you been drinking alcohol (beer, wine, or brandy wine) within the last three months?” Responses were given on an 8-point scale, from 1 = I have never been drinking alcohol to 8 = 6–7 times a week. Smoking was measured at ages 13, 14, 15, 16, 18, and 19 with the item: “How often do you smoke?” Responses were given on a 4-point scale, from 1 = not at all to 4 = every day. All variables in this study were reverse coded so that higher scores indicate a greater magnitude (all scales are shown in reversed order above). To simplify our analyses, we averaged the available observed scores across all time points during adolescence for each health behavior for each participant. We believe this was justified because an early exploration of the health behavior variables, using latent growth models, indicated that the included health behaviors remained fairly stable during adolescence and that the vast majority of variance in these scores was explained by the latent intercept.

### Household income and parental education

Parental income was measured using household income for 1995, which was reported separately by both parents in one of six categories. Using the 01-01-1995 NOK-EURO exchange rate, these categories were: “Less than NOK 100,000 (approx. €11,899)”, “NOK 100–199,000 (approx. €11,900–23,999)”, “NOK 200–299,000 (approx. €24,000–34,499)”, “NOK 300–399,000 (approx. €35,500–46,399)”, “NOK 400–499,000 (approx. €47,400–59,299)”, and “NOK 500,000 or more (approx. €59,300 or more)”. Parental education was self-reported in the same year using one of six categories: “0 years of education after elementary school”, “1–2 years of education”, “3 years of education”, “Less than four years at university/college”, “More than four years at university/college”, and “Other”. Missing values and the last category, “other,” were replaced with the adolescents’ reports of parental educational attainment in one of six categories: “Elementary school (7 years)”, “Secondary (9 years)”, “Manual education”, “Office/trade education”, “Gymnasium”, and “University/higher education.”

Participants’ education at age 40 (2017) was measured using the same categories as reported for parental education, while participant (offspring) income at age 40 (2017) was measured using a 10-point scale: “Less than NOK 100,000”, “NOK 100–199,000”, “NOK 200–299,000”, “NOK 300–399,000”, “NOK 400–499,000”, “NOK 500,000–599,000”, “NOK 600,000–699,000”, “NOK 700,000–799,000”, “NOK 800,000–899,000”, and “NOK 900,000 or more.” These two variables (participants’ own education and income at age 40) were correlated with adult depressed mood scores at age 40 as an estimate of social inequality in adult depressed mood (i.e., the covariance between education/income and depressed mood).

### Control variables

Two of the most important confounders in the association between SES and depressive symptoms are age and gender [1]. Age had low variance in the present sample, and gender was controlled for by having respondents report their gender as either 1 = male or 2 = female at age 13. Moreover, to account for reverse causality (i.e., health selection) [58], adolescent depressed mood was controlled for (equivalent to pathway I in the APM). Depressed mood during adolescence was measured at ages 13, 15, and 18 using the same scale as for the primary adult outcome, and an average was computed for each respondent. As several studies also indicate that social support is an important predictor of depressive symptoms [59], we controlled for parental closeness and peer acceptance (equivalent to pathway III in the APM). This approach allows for a better measurement of the unique effects of health behaviors on adult depressed mood.

Parental closeness was measured at ages 13, 15, and 18 using the Parent-Adolescent Scale. Example items include: “My mother and I understand each other well” and “My father and I understand each other well” [60]. Items one to three were rated on a 6-point Likert Scale (1 = does not apply at all to 6 = applies exactly), and items four and five were rated on a scale from 1 = seldom or never to 6 = very often. Peer acceptance was measured at ages 13, 15, and 18 using two items rated from 1 = Incorrect to 6 = Very correct, and an average across time was computed for each respondent. An example item is: “My peers seem to like me.” Both scales demonstrated satisfactory psychometric properties in an earlier study [61].

### Statistical analyses

**Fig. 2:**
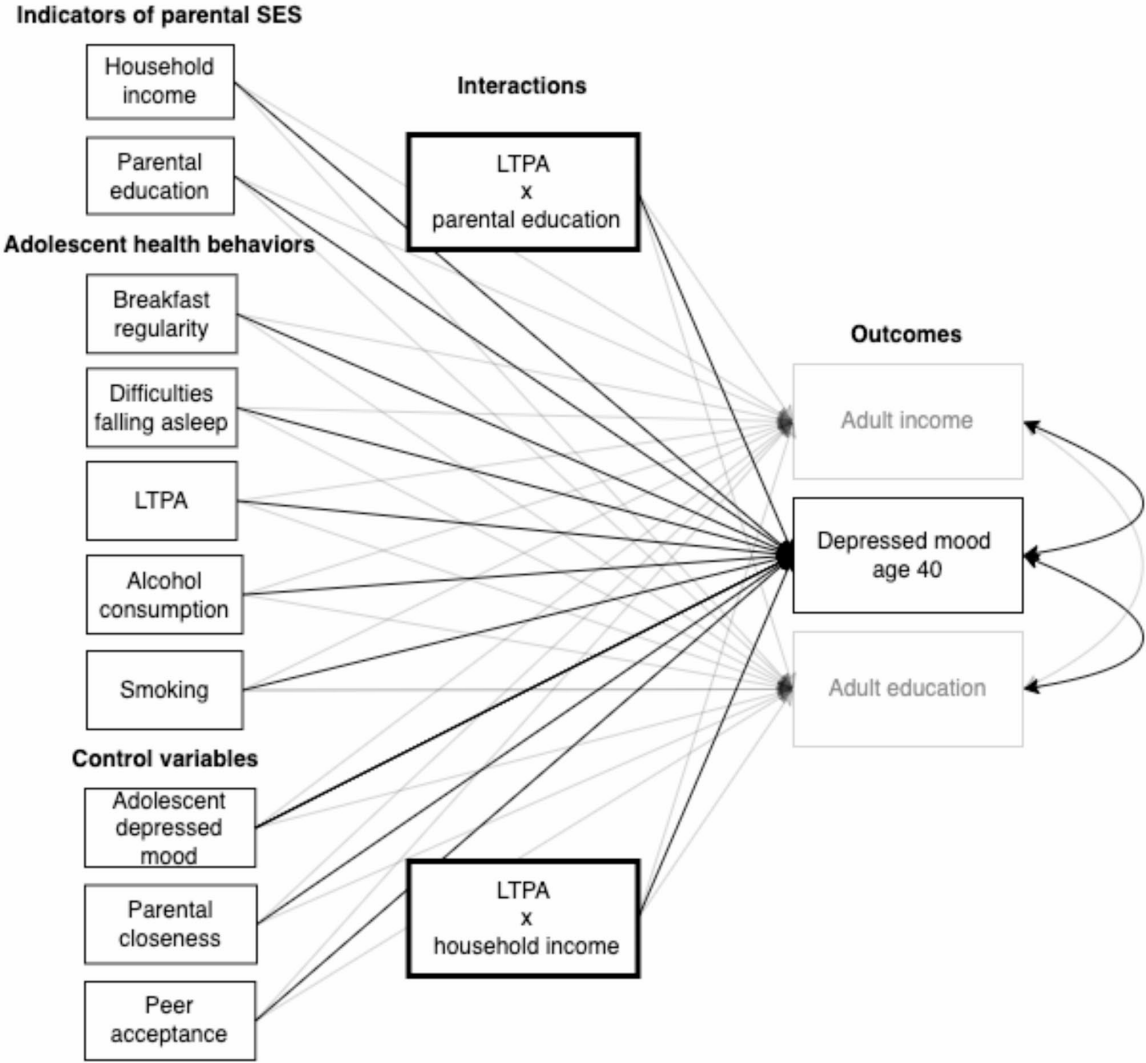
Model 6 A with interaction terms for parental education × leisure-time physical activity and household income × leisure-time physical activity. **Note**: Fig. 2 depicts Model 6 A with interaction terms for parental education × leisure-time physical activity and household income × leisure-time physical activity. These interaction terms correspond to socially differential vulnerability in the Adolescent Pathway Model. LTPA = leisure-time physical activity

The present study used SPSS v. 26 for dataset preparation (i.e., computing SES variables, averaging scores, coding missing data, etc.) and for the attrition analysis. Mplus v. 8.7 was used for the primary analyses. In Mplus, all analyses were conducted using Robust Maximum Likelihood Estimation (MLR) with Full Information Maximum Likelihood (FIML) to handle missing values. To address RQ1: To what extent do key adolescent health behaviors display socioeconomic differences (socially differential exposure)? We began by regressing each health behavior (breakfast regularity, LTPA, difficulties falling asleep, alcohol consumption, and smoking) on parental income and parental education separately. This approach allowed us to capture the distinct effects of each SES indicator. A final model then included both SES indicators simultaneously to assess their combined influence on adolescent health behaviors. Next, to address RQ2: Are adolescents from lower socioeconomic backgrounds more vulnerable to developing depressed mood in adulthood based on health behaviors during adolescence (socially differential vulnerability)? and RQ3: To what extent do adolescent health behaviors explain the relationship between adult depressed mood and adult education and income? we built a new model in six steps: Step One: We correlated adult depressed mood at age 40 with adult income and education at age 40. This was necessary to establish the baseline covariance between adult SES and depressed mood, which would later be tracked as predictors were added to the model to examine social inequality in adult depressed mood. Step Two: Gender, household income, and parental education were included as predictors of adult depressed mood, income, and education. This step helped assess the direct effects of SES on adult outcomes, while controlling for gender, an important confounder in the association between SES and depressive symptoms [1]. Step Three: Breakfast regularity, LTPA, difficulties falling asleep, alcohol consumption, and smoking were added as predictors of adult depressed mood, income, and education. This allowed us to estimate both the direct effects of these health behaviors on adult depressed mood and their role in accounting for the covariance between adult SES (income and education) and depressed mood. Step Four: Parental closeness and peer acceptance were included as control variables to test the robustness of the health behavior estimates. This step ensured that any effects observed were not confounded by social support during adolescence. Step Five: Adolescent depressed mood was added to account for baseline mental health status, ensuring that the effects observed were not merely due to pre-existing depressive tendencies during adolescence. Step Six (Models 6 A to 6E): We tested whether parental SES moderated the relationship between each health behavior and adult depressed mood (socially differential vulnerability, Mechanism D in the APM). Interaction terms were tested for each health behavior separately to avoid multicollinearity and ensure model parsimony (see Fig. 2 for Model 6 A). This step was essential for addressing RQ2, allowing us to explore differential vulnerability based on socioeconomic background. To test the robustness of our interaction models, we also ran them without including adolescent depressed mood and social support as control variables.

## Results

### Descriptive statistics

Our analytic sample consisted of *n* = 1109 (45.5% females). Descriptive statistics and correlations between study variables are reported in Table 1.

**Table 1:** Descriptive statistics and correlations for study variables

Variable	*n*	Min/Max	M	SD	1	2	3	4	5	6	7	8	9	10	11	12	13
1. Depressed mood (age 40)	449	1/6	1.63	0.83													
2. Parental education	968	1/5	2.58	1.18	− 0.14**												
3. Household income	615	1/6	4.29	1.24	− 0.08	0.40**											
4. Participant income (age 40)	448	1/10	6.44	2.13	− 0.26**	0.15**	0.07										
5. Participant education (age 40)	450	1/5	4.08	1.07	− 0.23**	0.35**	0.21**	0.23**									
6. Adolescent depressed mood	1061	1/6	2.38	0.91	0.32**	− 0.13**	− 0.18**	− 0.23**	− 0.16**								
7. Parental closeness	1065	1.5/6	4.45	0.88	− 0.22**	0.10**	0.13**	0.15**	0.089	− 0.37**							
8. Peer acceptance	1083	1/6	4.65	0.79	0.19**	0.09**	0.13**	0.18**	0.14**	0.31**	0.39**						
9. Difficulties falling asleep^1^	1052	1/4	1.88	0.50	0.10*	0.01	− 0.08	− 0.04	− 0.00	0.20**	− 0.13**	− 0.15**					
10. LTPA	1103	1/7	4.69	1.13	− 0.16**	0.06	0.15**	0.28**	0.14**	− 0.16**	0.16**	0.17**	− 0.13**				
11. Breakfast regularity	1093	1/4	3.47	0.79	− 0.19**	− 0.16**	0.14**	0.15**	0.15**	− 0.16**	0.18**	0.07*	− 0.08*	0.13**			
12. Smoking	1099	1/4	1.78	0.95	0.11*	− 0.09**	− 0.10*	− 0.08	− 0.21**	0.20**	− 0.17**	0.04	0.10**	− 0.18**	− 0.23**		
13. Alcohol consumption	1089	1/8	2.74	1.29	0.04	− 0.04	− 0.00	0.04	− 0.13**	0.14**	− 0.17**	0.07*	0.09**	− 0.11**	− 0.16**	0.61**	

Note. ^*^p <.05. ^**^p <.01. Note. n = no. of observations, M = mean, Min = Minimum value, Max = Maximum value, SD = Standard Deviation

^1^The scale was shortened to the first three response categories from 1993 and onwards

LTPA = Leisure time physical activity

### Attrition analysis

To examine attrition patterns, we categorized participants into respondents and non-respondents (coded as 1 and 0, respectively). Independent sample t-tests were conducted to assess differences in breakfast regularity, leisure time physical activity (LTPA), difficulties falling asleep, alcohol consumption, and smoking between participants who remained in the study and those who dropped out by 2017 (See Table 2). The results indicated that participants who reported higher levels of difficulties falling asleep and more regular breakfast consumption were more likely to continue participating in 2017 at age 40.

**Table 2:** Independent sample t-tests for responders and non-responders at age 40 (2017)

Participation	Yes		No												
	*n*	Mean (SD)	*n*	Mean (SD)	Mean difference		F-value		Two-tailed p-value			t- value		df	
Breakfast regularity	448	3.60(0.68)	645	3.40(0.90)	− 0.20***		44.48		*p* <.001			-4.36		1070.43	
LTPA	450	4.70(1.10)	635	4.70(1.20)	0.02		4.67		0.77			0.29		1028.55	
Difficulties falling asleep	440	1.94(0.44)	612	1.83(0.54)	− 0.11***		15.18		*p* <.001			-3.75		1035.70	
Alcohol consumption	449	2.73(1.17)	640	2.75(1.37)	0.02		14.95		0.76			0.29		1045.84	
Smoking	449	1.72(0.90)	650	1.82(0.99)	0.10		7.18		0.08			1.78		1017.87	

Note. Estimates in bold are significantly different from zero (*** *p* <.001, ** *p* <.01, * *p* <.05). LTPA = Leisure time physical activity

### The association between indicators of parental SES and health behaviors

When regressing health behaviors on parental education, the results showed that higher levels of parental education predicted lower levels of adolescent smoking (β = − 0.09, 95% CI [-0.15, − 0.03], *p* =.003) and higher levels of breakfast regularity (β = 0.16, 95% CI [0.10, 0.22], *p* <.001). Alcohol consumption (β = − 0.04, 95% CI [-0.10, 0.03], *p* =.286), leisuretime physical activity (LTPA) (β = 0.06, 95% CI [0.00, 0.13], *p* =.050), and difficulties falling asleep (β = 0.01, 95% CI [-0.06, 0.07], *p* =.824) were not associated with parental education. When regressing health behaviors on household income, higher levels of household income predicted lower levels of adolescent smoking (β = − 0.10, 95% CI [-0.18, − 0.02], *p* =.015) and difficulties falling asleep (β = − 0.10, 95% CI [-0.20, − 0.00], *p* =.044). It was also associated with higher levels of breakfast regularity (β = 0.15, 95% CI [0.06, 0.24], *p* =.001) and LTPA (β = 0.15, 95% CI [0.08, 0.23], *p* <.001). No association was found for alcohol consumption (β = − 0.00, 95% CI [-0.09, 0.08], *p* =.939). With the inclusion of both parental education and household income in the model, higher household income predicted higher levels of LTPA (β = 0.15, 95% CI [0.07, 0.24], *p* <.001), while higher parental education predicted higher breakfast regularity (β = 0.13, 95% CI [0.06, 0.20], *p* <.001). None of the other associations with health behaviors were significant. Smoking was not predicted by either parental education (β = − 0.07, 95% CI [-0.13, 0.00], *p* =.066) or household income (β = − 0.07, 95% CI [-0.16, 0.02], *p* =.126). Alcohol consumption was also not predicted by parental education (β = − 0.04, 95% CI [-0.12, 0.03], *p* =.234) or household income (β = 0.02, 95% CI [-0.07, 0.11], *p* =.630). Finally, difficulties falling asleep were not associated with parental education (β = 0.04, 95% CI [-0.04, 0.11], *p* =.346) or household income (β = − 0.08, 95% CI [-0.18, 0.03], *p* =.171). Adjusting for gender did not significantly change the associations.

### Adolescent health behaviors as predictors of adult depressed mood

As shown in Table 3, parental education was a weak but significant predictor of adult depressed mood, while parental income was not (Model 2). Among the health behaviors, higher levels of leisure time physical activity (LTPA) and greater breakfast regularity were associated with reduced adult depressed mood, whereas difficulties falling asleep were linked to higher levels of adult depressed mood (Model 3). These associations appeared relatively independent of parental education, as the point estimates for these health behaviors did not change significantly in Model 3. In Model 4, after controlling for the social support variables, none of the health behaviors remained significant. This finding persisted in Model 5, which included adolescent depressed mood, the only significant predictor in the full model. Additionally, the association between parental education and adult depressed mood became non-significant only in Model 5.

**Table 3:** Standardized estimates (β) of the effects of health behaviors on depressed mood at age 40 across models 1 to 5

	Model 1 (Correlational model)	Model 2 (with gender and parental SES indicators)	Model 3 (with health behaviors)	Model 4 (adjusted for parental and peer support)	Model 5 (adjusted for adolescent depressed mood)
Gender	**-**	0.04 [-0.06, 0.13]	− 0.02 [-0.11,0.08]	− 0.04 [-0.13,0.05]	− 0.05 [-0.14, 0.04]
Parental education	**-**	− 0.12* [-0.22,** − 0.03]**	− 0.11* [-0.20,**-0.13]**	− 0.09* [-0.18,**0.00]**	− 0.08 [-0.17, 0.01]
Household income	**-**	− 0.04 [-0.19, 0.11]	− 0.01 [-0.16,0.14]	0.01 [-0.14,0.16]	0.03 [-0.12, 0.17]
Smoking	**-**	**-**	0.07 [0.09,0.24]	0.06 [-0.10,0.22]	0.04 [-0.13, 0.20]
Alcohol consumption	**-**	**-**	− 0.05 [-0.18,0.08]	− 0.07 [-0.19,0.06]	− 0.06 [-0.19, 0.07]
LTPA	**-**	**-**	− 0.12* [-0.22,**0.02]**	− 0.10 [-0.21,0.01]	− 0.09 [-0.20, 0.01]
Difficulties falling asleep	**-**	**-**	0.10* [0.00,**0.21]**	0.07 [-0.03,0.01]	0.03 [-0.07, 0.14]
Breakfast regularity	**-**	**-**	− 0.15* [-0.29,**0.01]**	− 0.14 [-0.27,0.00]	− 0.10 [-0.25, 0.04]
Parental closeness	**-**	**-**	**-**	− 0.15** [-0.26,**-0.05]**	− 0.10 [-0.21, 0.01]
Peer acceptance	**-**	**-**	**-**	− 0.07 [-0.18,0.04]	− 0.02 [-0.13, 0.09]
Adolescent depressed mood	**-**	**-**	**-**	-	0.22*** [0.10,** 0.34]**
**Pearson correlations between indicators of adult SES and depressed mood**					
Adult depressed mood and adult education	− 0.23*** **[-0.34**,** − 0.13]**	− 0.20*** **[-0.30**,** − 0.09]**	− 0.17** **[-0.27**,**.-0.07]**	− 0.17** **[-0.26**,**-0.07]**	− 0.16*** **[-0.26**,** − 0.06]**
Adult depressed mood and adult income	− 0.26*** **[-0.36**,** − 0.16]**	− 0.24*** **[-0.35**,** − 0.14]**	− 0.21*** **[-0.32**,**-0.10]**	− 0.20*** **[-0.31**,**-0.09]**	− 0.19** **[-0.30**,** − 0.08]**

Note. Standardized estimates are presented with 95% confidence intervals in brackets. Estimates in bold are significantly different from zero (***p <.001, **p <.01, *p <.05). LTPA = Leisure time physical activity

### The relationship between adolescent health behaviors and adult depressed mood across different levels of parental SES indicators

No evidence was found for a moderating effect of parental SES on the association between adolescent health behaviors and adult depressed mood (see Supplementary Material 1, Table S1, for standardized estimates from models 6 A-6E). Of the tested interaction effects, only the interaction in Model 6 A between parental education and LTPA was statistically significant (β = − 0.82, 95% CI [-1.39, − 0.26], *p* =.004). However, an inspection of the conditional association between LTPA and adult depressed mood showed that this relationship was statistically significant only at ± 5 standard deviations from the average parental education level, indicating that the interaction had limited practical significance. Robustness checks, conducted without controlling for adolescent depressed mood and social support variables, yielded similar findings.

### The contribution of adolescent health behaviors to explaining social inequality in adult depressed mood

The correlations between adult income, adult education, and adult depressed mood were negative and moderately weak. Adolescent health behaviors explained 18% of the correlation between adult education and depressed mood, and 14% of the correlation between adult income and depressed mood (Model 3). However, results from Models 4 and 5 showed that adolescent health behaviors did not independently contribute to explaining social inequality in adult depressed mood.

### Discussion

The present study set out to investigate the impact of adolescent health behaviors on adult depressed mood within the APM framework. The research questions were: RQ1: To what extent do key adolescent health behaviors display socioeconomic differences (socially differential exposure)? RQ2: Are adolescents from lower socioeconomic backgrounds more vulnerable to developing depressed mood in adulthood based on health behaviors during adolescence (socially differential vulnerability)? RQ3: To what extent do adolescent health behaviors explain the relationship between adult depressed mood and adult education and income, as posited by the behavioral explanation of health inequalities?

With respect to RQ1, the findings showed some degree of socially differential exposure with higher household income predicting higher levels of LTPA, and higher parental education predicting higher levels of breakfast regularity. Direct effects of health behaviors on depressed mood further revealed that higher levels of LTPA and breakfast regularity predicted lower levels of adult depressed mood, while difficulties falling asleep predicted higher levels. However, these effects were not robust when adjusting for social support. In relation to RQ2, no socially differential vulnerability was indicated as none of the health behaviors were moderated by parental SES in their effects on adult depressed mood. Lastly, in relation RQ3, health behaviors were not found to account for any significant part of the covariance between adult depressed mood and indicators of adult SES (parental income and education) when adjusting for baseline adolescent depressed mood. Overall, these findings provide limited support for the social formation of adolescent health behaviors (i.e., socially differential exposure) and their role in contributing to social inequalities in adult depressed mood (i.e. the behavioral explanation).

In the present study, one of the significant findings on socially differential exposure was that the association between higher breakfast regularity during adolescence was associated with higher parental education. This has also been found in other studies - indicating that breakfast consumption is more common in families with higher levels of affluence [62]. This is, in part, due to the availability of breakfast foods [63], but socioeconomic background may also influence breakfast habits through mechanisms such as parental rules for breakfast consumption, adolescents modeling parental behavior, and co-participation in meals—all of which have been linked to levels of breakfast consumption [63, 64].

The other significant finding on socially differential exposure was that higher parental household income was associated with higher LTPA. In Norway, organized LTPA is more or less exclusively offered through sports clubs with membership fees that are costly for the average family [65]. Importantly, sports clubs in Norway are also heavily dependent upon parents volunteering and assisting as coaches [66]. In Norway, this has also been found to introduce neighborhood-based differences in LTPA based on parental resourcefulness and availability of organized sports activities [67]. This finding is important, because even though the present study found SES-differences in adolescent LTPA, but no effects of LTPA on adult depressed mood, a UK study found that physical activity levels at age 16 reduced the incidence and risk of incidence elevated depressive symptoms in both mid-adulthood and late adulthood [11]. This study also adjusted for baseline levels of depressive symptoms in earlier years. Similar studies also report long-term effects of adolescent physical activity on adult depressive symptoms with baseline adjustment [12, 68]. This speaks to the importance of reducing socioeconomic barriers to physical activity in adolescence.

The absence of socially differential exposure for other health behaviors investigated in this study (adolescent alcohol consumption, smoking, and difficulties falling asleep) aligns with the notion of equalization but is surprising given earlier research [16, 29–31, 52, 69–73]. It may be that some health behaviors are more influenced by family socioeconomic status (SES), whereas others are more affected by peers, as suggested by West (1997) [74]. For example, alcohol consumption [75] and smoking [76] often occur in peer settings and are related to factors such as peer pressure [77].

The lack of SES-based disparities in difficulties falling asleep is somewhat unexpected because lower socioeconomic status is often linked to more dysfunctional family environments [78–80], which are negatively associated with sleep quality [81]. It is also suggested by mechanism A in the APM that lower SES is associated with greater family dysfunction, potentially contributing to longer sleep latency (i.e., difficulties falling asleep) [17]. Given this, we would expect adolescents from lower SES backgrounds to experience more sleep difficulties. Still, studies on social disparities in sleep duration have produced mixed findings overall [82–87]. These inconsistencies may partly be due to varying ways in which SES and sleep problems are conceptualized and measured [88]. Therefore, further research is needed, using a broader range of indicators to explore multiple dimensions of both SES and sleep issues.

In the present study, socially differential vulnerability was also investigated. The findings revealed no differential associations between health behaviors and adult depressed mood based on socioeconomic background in adolescence. Specifically, the effects of health behaviors on depressed mood were not moderated by parental socioeconomic status (SES), indicating that there were no more aversive effects for adolescents from lower SES backgrounds (Mechanism D in Fig. 1). This aligns with the findings by Akasaki, Ploubidis [41], who observed that parental occupational ranking did not moderate the effects of clustered adolescent health behaviors on psychological distress in mid-life. Several possible explanations could account for this lack of differential vulnerability. First and foremost, this finding should be considered in light of the minimal socioeconomic differences observed in adolescent health behaviors, or socially differential exposure. If adolescent health behaviors are largely equalized across socioeconomic groups, they would not be expected to contribute to an increased allostatic load or higher levels of depressed mood in adulthood for those from disadvantaged backgrounds. Additionally, since this study focuses on Norwegian adolescents, it could be argued that the Nordic welfare state may have mitigated the differential effects of socioeconomic background on depressive symptoms [47]. Although finding directly comparable studies in terms of population and design is challenging, existing literature generally suggests that welfare regimes do not significantly buffer against socioeconomic inequalities in health outcomes [89, 90]. Another explanation could be that individual attributes—such as personality traits and cognitive ability—might play a more crucial role in forming social disparities in health, as opposed to traditional indicators like parental education and income [91]. That is, in highly developed welfare societies like Norway, individual characteristics may exert a stronger influence on future health outcomes due to greater social mobility [92, 93]. However, the present study lacked adequate data on individual characteristics to investigate this hypothesis.

In addressing RQ3, the present study examined the extent to which adolescent health behaviors contribute to explaining social disparities in adult depressed mood, specifically the covariances among adult income, education, and depressed mood. We found that the contributions of health behaviors were not robust when adjusting for baseline adolescent depressed mood. There are few studies available for direct comparison with this finding. While health behaviors are well-documented predictors of inequalities in physical health outcomes, such as premature mortality and disability [25, 47], fewer studies have focused on their influence on mental health outcomes, particularly depressive symptoms.

Finally, the findings of the present study suggest that homotypic continuity—the persistence of the same mental health issues over time—plays a significant role in perpetuating social inequalities in depressed mood from adolescence into adulthood. This aligns with another Norwegian study, which found no association between adolescent health behaviors (such as diet, physical activity, and sleep) and adult depression when adjusting for adolescent psychological distress, which emerged as the strongest predictor of adult depression [94]. This pattern is further supported by multiple studies demonstrating homotypic continuity in depressive symptoms [95–97], indicating that early mental health difficulties may have a lasting impact, regardless of other factors such as health behaviors. This finding can be interpreted as supporting the health selection hypothesis [98], which, in the context of this study, suggests that adolescent depressed mood can partly explain both socioeconomic achievement and the persistence of depressed mood into adulthood. Thus, contrary to the health behaviors pathway outlined in the APM framework, the behavioral explanation (i.e., health behaviors as a cause of SES-based health inequalities) may not play a significant intermediary role between SES and health in producing socioeconomic inequalities in depressed mood.

### Strengths and limitations

To the authors’ knowledge, the present study is one of the first to investigate the effects of adolescent health behaviors on adult depressed mood from adolescence to mid-adulthood using the APM framework. It is also one of the first studies to explore socially differential vulnerability to depressed mood, as well as the contribution of health behaviors in explaining social disparities in adult depressed mood. However, several limitations should be noted. Firstly, when estimating social differential exposure, we did not control for childhood socioeconomic status (SES) or stressful exposures during childhood, which might have confounded our measure of the association between adolescent SES and adolescent health behaviors. Secondly, our selection of health behaviors was rather limited; for example, we lacked sufficient data on risky sexual behavior [99], abuse of illegal substances [100], problematic/pathological gaming [101], and the nutritional quality of meals [102]—all of which could have been relevant to depressive mood and might have yielded different findings. Recent research has also shown that several newer health behaviors, such as screen time [103] and social media use [104], are linked to depressive symptoms and related mental health outcomes; therefore, future studies could benefit from including these factors. Moreover, health behaviors were measured using single items, which may have introduced measurement error and recall bias. However, using multiple-item scales to measure health behaviors is not necessarily preferable [105], as these behaviors are generally less complex than latent constructs (e.g., personality, intelligence). Additionally, employing extra scales would have resulted in a higher respondent burden, potentially leading to increased attrition and reduced sample sizes at later time points. This could introduce additional statistical limitations, such as reduced power and increased bias. Relatedly, while breakfast regularity had a high mean (3.47) on a 1–4 scale, suggesting a ceiling effect, the standard deviation of 0.79 indicates substantial variation among respondents. In light of this variability, including individual characteristics (i.e., genetics, personality, cognitive ability, etc.) could have provided more precise estimates of the role of adolescent health behaviors on adult depressed mood. Furthermore, the significant differences in difficulties falling asleep and breakfast regularity suggest that attrition may not be completely random. Specifically, the patterns indicate the possibility of a Missing Not at Random (MNAR) mechanism, as those with more difficulties falling asleep and less regular breakfast consumption are more likely to have dropped out. Additionally, female participants from households with higher incomes and higher parental education levels were more likely to continue participating until the latest time point in 2017. This suggests that the missing data could be systematically related to the variables of interest, potentially biasing the results. In addition, this indicates potential biases in the representativeness of the sample, as participants who remained in the study may not fully reflect the broader population, particularly in terms of socioeconomic background and health behaviors. Finally, it is important to test the APM on contemporary datasets that reflect modern adolescent health behaviors. This should be done in a manner that facilitates causal modeling, enabling a more in-depth exploration of the mechanisms and pathways involved.

## Conclusion

Findings from the current study indicate minimal socioeconomic differences in adolescent health behaviors, suggesting that these behaviors do not significantly contribute to social inequalities in adult depressed mood. This provides limited support for the behavioral explanation of health inequalities within the framework of the APM. Instead, adolescent depressed mood emerges as the strongest predictor of adult depressed mood, underscoring its role in early intervention and prevention efforts.

## Electronic supplementary material

Below is the link to the electronic supplementary material.

Supplementary Material 1

## Data Availability

The authors agree to make data, materials and scripts supporting the results or analyses presented in the paper available upon reasonable request.
