# Supplementary material for: Socioeconomic differences in adolescent health behaviors and their effect on inequalities in adult depressed mood: findings from a 27-year longitudinal study
Source: BMC Psychiatry. 2025 Apr 10;25:364. doi: 10.1186/s12888-025-06679-6 (PMC11987293; doi:10.1186/s12888-025-06679-6)
Supplement: Supplementary file 1 — Supplementary Material 1 [file 12888_2025_6679_MOESM1_ESM.docx]

**Table S1. The association between adolescent health behaviors and adult depressed mood with interaction terms by parental education and household income**

|  | **Interaction by levels of**  **parental education** | **Interaction by levels of**  **household income** |
| --- | --- | --- |
| LTPA (Model 6A) | **-.82**[-1.39,-.26]** | .51[-.32,1.34] |
| Smoking (Model 6B) | -.05[-.45,.35] | .03[-.56,.62] |
| Alcohol consumption (Model 6C) | .06[-.31,.43] | .21[-.55,.96] |
| Difficulties falling asleep (Model 6D) | .30[-.26,.86] | -.31[-1.23,.60] |
| Breakfast regularity (Model 6E) | .28[-.65,1.21] | .47[-.52,1.45] |

Note. Standardized estimates are presented with 95% confidence intervals in brackets. Estimates in bold are significantly different from zero (*** *p* < .001, ** *p* < .01, * *p* < .05). Each pair of interaction terms were tested together in separate models for each of the health behaviors. LTPA = Leisure time physical activity.
